# The Relationship Between SNS Usage and Disordered Eating Behaviors: A Meta-Analysis

**DOI:** 10.3389/fpsyg.2021.641919

**Published:** 2021-08-02

**Authors:** Juan Zhang, Yihui Wang, Qianru Li, Chenggang Wu

**Affiliations:** ^1^Faculty of Education, University of Macau, Macau, China; ^2^Center for Cognitive and Brain Sciences, University of Macau, Macau, China; ^3^School of Education, Shanghai International Studies University, Shanghai, China

**Keywords:** social networking sites, eating disorder, social media, BMI, disordered eating behaviors

## Abstract

Social Networking Sites (SNSs) are common tools with which modern people share their lives and establish social relationships. However, some studies have found SNSs to be associated with eating disorders, although other have identified no connection between the two. To explore the interaction between SNSs and eating disorder behaviors, this study aimed to comprehensively synthesize previous studies using meta-analysis methods. Based on selection criteria, there were 87 effect sizes from 22 studies. After analysis using a three-level random-effects meta-analysis model, a positive correlation between the use of SNSs and irregular eating behaviors was found, *r* = 0.09 (95% CI: 0.06, 0.11; *p* < 0.001). In addition, by analyzing potential moderators, body mass index (*r* = −0.032; 95% CI: −0.058, −0.006; *p* = 0.019), survey methods, and sample sources was discovered could alter the relationship between SNSs and disordered eating behaviors. Specifically, there was a significantly larger association between SNSs results obtained by paper and pencil surveys and disordered eating behaviors (*r* = 0.114; 95% CI: 0.081, 0.147; *p* < 0.001) than that between SNSs results obtained by online surveys and disordered eating behaviors (*r* = −0.055; 95% CI: −0.102, −0.007; *p* < 0.01). University students showed a larger correlation between SNSs and disordered eating behavior than other samples (*r* = 0.089; 95% CI: 0.049, 0.129; *p* < 0.001). Overall, this meta-analysis confirms that the excessive use of SNSs is associated with an increased risks of disordered eating behaviors. It is hoped that this study can provide a reference for the management and intervention of dietary behaviors related to social networks in the future.

## Introduction

Eating disorders (EDs) are recognized mental illnesses characterized by irregular eating habits and abnormal concerns about body weight and shapes. Such disorders are chronic, difficult to recover from, prone to relapse and often have serious sequelae (Brownell and Walsh, 2017; Rodgers et al., 2018; Galmiche et al., 2019). Many studies confirmed that EDs make people more vulnerable to psychiatric illnesses such as anxiety and depression, as well as bodily diseases such as diabetes and obesity (Fairburn et al., 2000; Johnson et al., 2001; Schmidt et al., 2016). People with EDs experience a reduced quality of life compared with those suffering from other mental illnesses and physical health conditions (Jenkins et al., 2011a). In severe cases, EDs have been found to be related to suicide and other forms of premature mortality (Favaro and Santonastaso, 1997; Bulik et al., 1999). Currently, millions of people suffer from EDs, and the general probability of being affected by ED-related symptoms in one's lifetime is 10% (Schaumberg et al., 2017). Moreover, research has shown that the effects of EDs may reduce family cohesion, increase financial and psychological pressure on family members, and increase psychological risk factors among peers, including the risk of suffering from EDs (Hillege et al., 2006; Keel and Forney, 2013). Disordered eating behaviors represent the core symptoms of EDs. They may consist of implicit attitudes (e.g., eating, weight, and shape concerns) or explicit behaviors (e.g., binge eating, emotional eating, dietary restriction) (Fergus et al., 2019). Whether among young people or adults, irregular eating behaviors are associated with continuous stress, anxiety and other psychological problems, as well as physical health problems such as severe weight fluctuations, which may seriously impair their daily lives (Jenkins et al., 2011b; Neumark-Sztainer et al., 2012; Kärkkäinen et al., 2018). Fueled by these research significances, it is of special interest to examine the risk factors associated with disordered eating behaviors.

Numerous studies have been conducted to investigate the causes of disordered eating behaviors. In this regard, SNSs, as online communication platforms, have become a novel research area of particular interests (Brandtzæg and Heim, 2009). SNSs are among the most popular websites, as identified by statistical websites (Internet Live Stats, 2021). Studies have revealed that, although SNSs bring utility to daily life, their improper use appears to also bring about various problems. Excessive dependence on SNSs can diminish people's satisfaction with their lives, and may increase the likelihood of some people, notably teenagers, feeling depressed and lonely (Spraggins, 2009; Valkenburg and Peter, 2009; Das and Sahoo, 2011). Although several studies have found that concerns about body shape and disordered eating attitudes are positively related to the length of time spent on social media, the results of associated studies have not been consistent (Smith et al., 2013; Mabe et al., 2014; Holland and Tiggemann, 2017). For instance, some discrepancies have been found in the reported relationships between dietary behaviors and different types of SNSs (Kim and Chock, 2015; Blassingame, 2020b).

Although no previous meta-analysis has been conducted in this area, there was one systematic review of 20 articles published before 2016 (Holland and Tiggemann, 2016). This review not only focused on the relationship between SNSs and EDs but also on the relationship between SNSs and body image. It therefore only covered three studies that explicitly discussed the relationship between SNSs and EDs. Although the review concluded that an increase in SNS use was associated with disordered eating behaviors, its results did not indicate the extent to which these two variables were linked.

Since 2018, the related studies have risen sharply in number, and they have begun to explore the interaction between SNSs and EDs in more detail. Some have confirmed the findings of the review mentioned above (Teo and Collinson, 2019; Rodgers et al., 2020), but others have found that the use of SNSs was not directly related to disordered eating behaviors or attitudes (Howard et al., 2017; Cohen et al., 2018; Griffiths et al., 2018a; Blassingame, 2020b). To obtain a better understanding of the relationship between SNS usage and disordered eating behaviors, it is therefore necessary to conduct an up-to-date meta-analysis.

### SNS Usage

#### Development of Social Networking Sites

SNSs are defined as websites or applications located on the Internet that provide individuals with platforms for displaying and sharing their personal lives and interacting with others through provided functions such as comments, likes, and reposts (Perloff, 2014). Compared with traditional media, the emerging Internet-based media provide users with richer information and more diverse communication platforms (Xue and Yu, 2017). SNSs treat users as active participants, not as passive recipients of content gathered from related organizations. They provide users with a great deal of freedom, allowing them to exchange views and develop relationships with each other (Sharma and Verma, 2018). The most popular SNSs in the market, such as Facebook, Twitter, Instagram, and Myspace not only have these qualities, but have also been continuously improved to provide users with a stronger sense of immersion, intimacy, and belonging when they interact with their virtual social circles (Bullas, 2014). As of June 2019, the number of people using SNSs accounted for 72% of the world's population, and this number is increasing every year (Social Networking Fact Sheets, 2019).

#### Measurement of SNS Usage

Since the focus of this study is on the duration, frequency, and intensity of SNS use, most of the questionnaires it examines are adapted from questionnaires previously used to measure other network activities such as Internet use and Facebook use (Tiggemann et al., 2013; Vanden Abeele et al., 2018). Some questionnaires required participants to answer questions explicitly about the frequency of their use of social software; others asked them to circle a number on the Likert scale that they thought accurately represented this frequency (Griffiths et al., 2018a).

### SNS Usage and Disordered Eating Behaviors

Although the way that people participate in SNS activities is completely different from their use of traditional media, research has shown that they might still be as influenced by SNSs as they are by traditional media (Holland and Tiggemann, 2016). For example, studies have revealed that when young females use social media, the pictures they see generally promote slimness as an ideal form of beauty (Tiggemann and Miller, 2010; Fardouly et al., 2015; Brown and Tiggemann, 2016). Previous studies have shown how teenagers' comments and reposts of content containing body stereotypes might induce others to subconsciously approve of this aesthetic and extend its influence in the community (Boyd, 2014). In addition, SNSs may lead people to compare their appearances, body shapes, and affluence. Studies have found that some adolescent girls tend to post their selfies and “outfit-of-the-day” photos on social networking sites, hoping to prove that they are more appealing than their peers through the comments and compliments they receive (Kaplan and Haenlein, 2010).

Two theoretical frameworks are relevant to explaining the possible relationship between EDs and SNSs. According to sociocultural models, people who interact closely with individuals influence their views about weight and the body (Stice, 1994). These models identify the social and cultural environment work as the most influential and pervasive forces encouraging individuals to promote and pursue ideals of slimness (Rodgers, 2016). In most cases, the figures presented on social media are slim, slender, toned and muscular, which most societies and cultures believe to be the ideal body shape (Carrotte et al., 2017). People struggle to attain this ideal, sometimes under heavy psychological pressure, as not everyone can achieve an idealized body shape, even after heavy exercises and strict control of energy intake (Stice, 1994; Hargreaves and Tiggemann, 2003; Keery et al., 2004; Ata et al., 2007). However, as human often show an instinctive desire to participate in social comparison behaviors automatically and spontaneously (Gilbert and Malone, 1995; Tiggemann, 2012), people tend to show an idealized version of themselves on social media (sometimes an unreal version) to gain recognition from others and from their cultural environment generally. Researchers have found correlations between social interactions in social networks and certain adverse outcomes, not only in terms of personal psychological features (self-esteem, body image, eating habits), but also affecting social relationships (a sense of belonging to the community, sense of happiness, and the ability to get along with others) (Davidson and Cotter, 1991; Obst and Stafurik, 2010). In particular, teenagers who frequently engage in comparisons with peers, or who often request reviews from others on Facebook, are found to be more likely to suffer from eating disorders such as binge eating and overeating (Smith et al., 2013).

Another widely-accepted theoretical basis for the relationship between EDs and SNSs is “self-objectification,” by which individuals commoditize their own value, and believes that the value of their personal identity is derived from the use and consumption of their body and its appearance to others (Fredrickson and Roberts, 1997). People may freely post any content that meets social media requirements on SNSs. This freedom has been found to indirectly encourage people (especially young women) to pay greater attention to their appearance than their feelings (Conger and Singg, 2020). Each time users employ the regular functions of SNSs (such as likes and reposts), they may deepen their internalization of a materialized conception of themselves (Chua and Chang, 2016). Research has demonstrated that such a pathological perception of the self is often related to body shame, and may predict an increased likelihood of depression, anxiety, and eating disorders (Przybylski et al., 2013; Beyens et al., 2016; Bell et al., 2018; Ramsey and Horan, 2018). Specifically, Choma et al. (2009), who investigated the self-conception and eating behaviors of female college students, found that body shame contributed to the impact of self-objectification in creating disordered eating. It therefore follows that SNS use may contribute to both self-objectification and body shame, and thus contribute to higher levels of disordered eating behaviors.

### Potential Moderators

In view of the sociocultural models, the objectification theory and the varying results summarized above, certain potential moderators should be considered when analyzing the data, to gain a thorough understanding of the relationship between SNS usage and disordered eating behaviors. These moderators are discussed below.

#### Type of SNS

There are several types of SNS, each with unique designs and functions. Image-centric social media (hereinafter referred to as image-based SNS) are social media whose functions are mainly based on the use of photography and other images. Examples include Instagram, Snapchat and Facebook (Rodgers and Melioli, 2016; Griffiths et al., 2018a). These may be contrasted with social media such as WordPress, whose users are not necessarily expected to display images (Griffiths et al., 2018b). Studies have indicated that, by promoting mutual comparisons among peers, the use of image-based SNSs increases the likelihood that individuals will acquire negative body images (Cohen et al., 2017; Hendrickse et al., 2017; Santarossa and Woodruff, 2017; Marengo et al., 2018). Thus, it may be reasonably inferred that the extent to which a certain SNS is designed based on images may contribute to its users' vulnerability to eating disorders.

#### Publication Time

Social media have developed rapidly, and new features or entirely new platforms may be appear over a short time (Bowman and Clark-Gordon, 2018). At the same time, the popularity of social media platforms is constantly shifting (Wilksch et al., 2019). A Finnish study found that between 2008 and 2016, the time people spent on SNS use increased and the purpose of their SNS use became more diverse, as did the types of their interactions on SNSs (Koiranen et al., 2019). We therefore infer that publication time is a potential moderator, as growing and widening SNS usage may affect the interaction between SNS and disordered eating behaviors.

#### Sex

A study pointed out that men and women display different habits in their use of SNSs, and that women tend to post more photos on social platforms, whether selfies, group photos or food photos (Wilksch et al., 2019). People's choices of SNSs may also be related to sex. For example, it was found that teenage girls are more likely to have Instagram and Tumblr accounts than teenage boys (Vannucci and Ohannessian, 2019). Furthermore, although the phenomenon of excessive use of SNSs leading to diet-related problems may be observed in both sexes, a study conducted by Ho et al. (2016) found that females' comparisons with friends and celebrities on SNSs showed a significantly closer relationship to body image dissatissfaction and the drive for slimness than those of males. These respective findings indicate that gender may affect the interaction between SNS use and disordered behaviors.

#### Region

Given the difference in political system between China and the West, the phenomena of social comparison in Chinese and Western societies may also differ, as the socialist environment places less emphasis on the individual (Hofstede, 1984; Bandura, 1995; Sedikides et al., 2003). Moreover, different cultures have different traditional aesthetics (Jankowiak et al., 2008). This may explain why, for example, research has shown that compared with Asian adolescents, American adolescents have a deeper internalization of the concept that “beauty is thin” (Leung et al., 2004; Marsh et al., 2007; Klaczynski and Felmban, 2019). Thus, it should be expected that studies on this subject from different regions may well provide different findings.

#### Age

People of different ages have different life priorities (Erikson, 1994), which might cause adults and adolescents to have different levels of dependence on social media. According to Australian research statistics from 2017, Australian aged between 14 and 17 spend on average half an hour more on social media every day than adults (Australian Psychological Society, 2017). In addition, young people are generally quicker to master new concepts and tools (such as new SNSs) and are more enthusiastic about using SNSs to conduct relationships with others (Kaur et al., 2016; Dhir and Tsai, 2017). Hence, age as a moderating factor in SNS usage may determine individual differences in disordered eating behaviors.

### Present Study

To the best of our knowledge, no previous meta-analysis has explored the combined effect of SNS usage and disordered eating behaviors. Given that meta-analysis can quantitatively summarize previous findings (e.g., the correlations between variables) with a large sample size, and can further speculate on the factors that might have affected the relationships between variables by moderator analysis, it is expected that the current meta-analysis will fill the research gap and enhance understanding of the literature on the relationship between disordered eating behaviors and SNS usage. First, this study aimed to collect all the relevant and accessible studies that were conducted before 2020. Second, this research analyzed many potential moderators in order to better explain the inconsistencies between previous research findings. A three-level random-effects meta-analysis model was adopted, which is suitable for obtaining a more accurate evaluation of the overall effect size in a large body of research (Van den Noortgate et al., 2013).

It was hypothesized that individuals experiencing a high intensity or duration of SNS usage would be more likely to exhibit disordered eating behaviors. However, since the results of previous studies did not reach a broad consensus on this subject, further hypothesize could not be on the influence of the moderators on the results of the current study.

## Method

### Literature Search and Study Selection

Related studies were searched and retrieved from five databases (PsychINFO, PubMed, Web of Science, Communication and Mass Media Complete, and ProQuest Dissertations) on July 18th, 2020. The following search keywords for social network usage and disordered eating were chosen: (“social media” OR “social networking sites” OR “SNS” OR “Twitter” OR “Facebook” OR “Weibo” OR “Instagram”) AND (“eating” OR “disordered eating” OR “eating disorder”). In addition, a manual search was performed of the reference list in the identified articles to find any other relevant research. It is worth noting that Google Scholar was selected as the fourth resource because it can span multiple disciplines, so that it can be used as a final check to ensure that all articles that meet the current inclusion criteria are captured. Considering the rise of social media, only documents published after 2010 were selected. In fact, no document exceeding this time limit during the search was found. This meta-analysis also aimed to find out the relationship between SNS and disordered eating behaviors in existing studies.

After the initial search, 480 articles were found. The following criteria were applied to screen the 480 articles:

(a) written in English;
(b) published in journals or dissertations;
(c) reported the correlation between social network usage (intensity or frequency) and disordered eating behaviors (r), which had to be a primary goal of the studies.

Only the articles that met the above three criteria were selected.

### Coding of Study Features

The following information was retrieved from the selected studies: (1) name of the first author; (2) publication year; (3) age; (4) Body mass index (BMI); (5) percentage of males; (6) percentage of college degree; (7) percentage of white; (8) publication type (dissertation or journal article); (9) region (Western or Eastern); (10) survey methods (paper-and-pencil or online); (11) sample source (university, children and adolescent, clinical or other); (12) SNS type (image-based, non-image-based or general); (13) SNSs usage (duration: time spent on SNSs; frequency: number of times of SNSs usage in a certain period; intensity: integration of SNSs into daily life); (14) type of disordered eating behavior (combined disordered eating behavior, binge eating, driving for thinness, bulimia or dietary restraint); (15) measure of eating (EAT-26, Project Eat III- Eating Behavior Questions, Eating Disorder Inventory, Dutch Eating Behavior Questionnaire, The EDE-Q or others; (16) correlations (*r*) between SNS and disordered eating behaviors.

To establish internal encoder reliability, two independent coders coded three articles randomly selected from 22 articles. After two rounds of coding, all coders achieved acceptable inter-coder reliability (the Cohen Kappa range of all variables in the coding scheme was 0.85–0.87). They then independently coded the remaining 19 articles and reached an absolute consensus of 95%. The coders resolve any differences through discussion to obtain the final coding result.

### Quality Appraisal

Quality assessment was performed by Q.L and Y.W independently. Disagreement about scores was resolved through discussion between the two authors. The quality of the studies included in the meta-analysis was retrieved and adapted from the previous studies (He et al., 2019, 2020). The adapted tool contained six items: the sampling method, the response rate of the study, the validity of the measurement tool, the source of data, the validity and reliability of examination of SNS usage, and the relationship between SNSs and disordered eating behaviors. Six items were “yes-or-no” questions with a score of 1 for yes and 0 for no. Appraisal scores were obtained by dividing the total score by the total number of items (six), then transforming the fraction into a percentage. In addition, the requirement for the measure of eating pathology relied on those assessments with reliability and validity, including EAT-26, Project EAT III-Eating Behavior Questions, Eating Disorder Inventory, Dutch Eating Behavior Questionnaire and the EDE-Q. This meta-analysis not only included diagnostic samplings, but also other samplings which had not been confirmed as diagnostic patients. Finally, the percentage of appraisal scores in this study was 81.1%, indicating good methodological quality.

To prevent the quality of a selected article from affecting the results, which was analyzed as a categorical moderator in the subsequent analysis (the first category is articles with a quality of 80% and above, and the rest are in the second category). The results show that the quality of the article does not affect the results of this study [*F*_(df1=1,df2=84)_ = 3.631, *p* = 0.060].

### Analysis Plan

As this was a cross-sectional analysis, all data analyses were performed with the R 4.0.0 (R Core Team, 2020) package of metaphor (Viechtbauer, 2010). Raw correlations were converted into Fisher's Zr, since estimate biases would be produced because variance closely relies on the criterion. The converted values were applied in the following analyses. However, Fisher's *Zrs* were back-transformed to correlation coefficients when reporting the results.

The outliers were inspected through the *altimeter* package with the function “meta-outliers” (Lin et al., 2017). A study would be considered as an outlier if the standard residual exceeded 3 (Viechtbauer and Cheung, 2010).

The heterogeneity was assessed by the Cochrane *Q* statistics test, which is a commonly-used index for probing the presence of unexplained heterogeneity (Higgins et al., 2003). Publication bias was examined through Begg's rank correlation test (Egger et al., 1997) and the symmetry of the funnel plot (Duval and Tweedie, 2000).

To avoid dependence problems such as effect sizes, observations, and error terms which are dependent and correlated if they are from the same study, a multilevel meta-analysis was used (Van den Noortgate et al., 2013). The three-level random-effects meta-analytic model was used to discompose variance in different sources. A sample of subjects for each experiment (Level 1), effect sizes within studies (Level 2), and effect sizes changed between studies (Level 3).

## Results

### Description of Studies Selected

**Figure 1:**
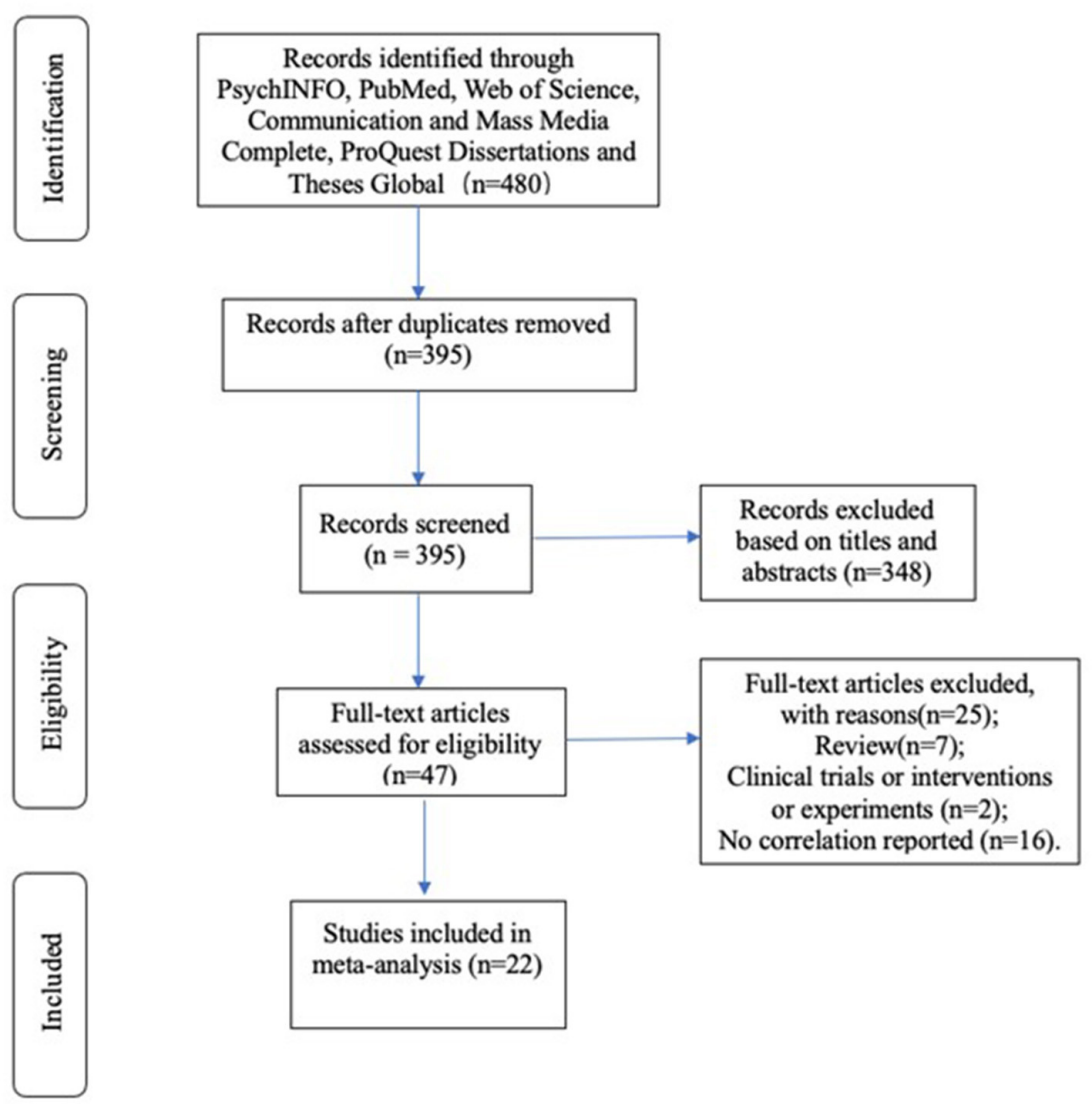
PRISMA flowchart.

Figure 1 shows a PRISMA flowchart (Hutton et al., 2015) illustrating the procedure for choosing the studies for this meta-analysis. In the searching process, five databases were used to find out appropriate studies. A total of 480 articles were obtained in the initial search. After screening for duplicates, 395 articles were left. In addition, 47 articles were selected through reading abstracts and matching criteria. Eventually, only 22 articles available for full-text review were included in the meta-analysis.

All studies included in this meta-analysis were published between 2010 and 2020. There were 29 independent sample sizes and 87 effect sizes in all 22 studies. A total of 13,301 samples were covered. The sample size of males was 5,031 (37.82% of the total), and 8,270 females. The average age of the sample was between 11.19 and 30.53, and the average BMI varied from 18.92 to 24.69. There were 73 effect sizes obtained from Western and 14 from Eastern. Moreover, 87 effect sizes were reported on the relationship between SNS and disordered eating behaviors (51.72% of the total effect size). Please refer to Supplementary Data Sheet 1 in Supplementary Material for the coding of this research.

### Quality Assessment

Samples, research methods, and data processing for the chosen articles were examined (see Appendix A for quality assessment standards). Overall, the included studies' methodological appraisal scores ranged from 66.7 to 100%, which indicated that all studies were of good methodological quality.

### Outlier Detection

**Figure 2:**
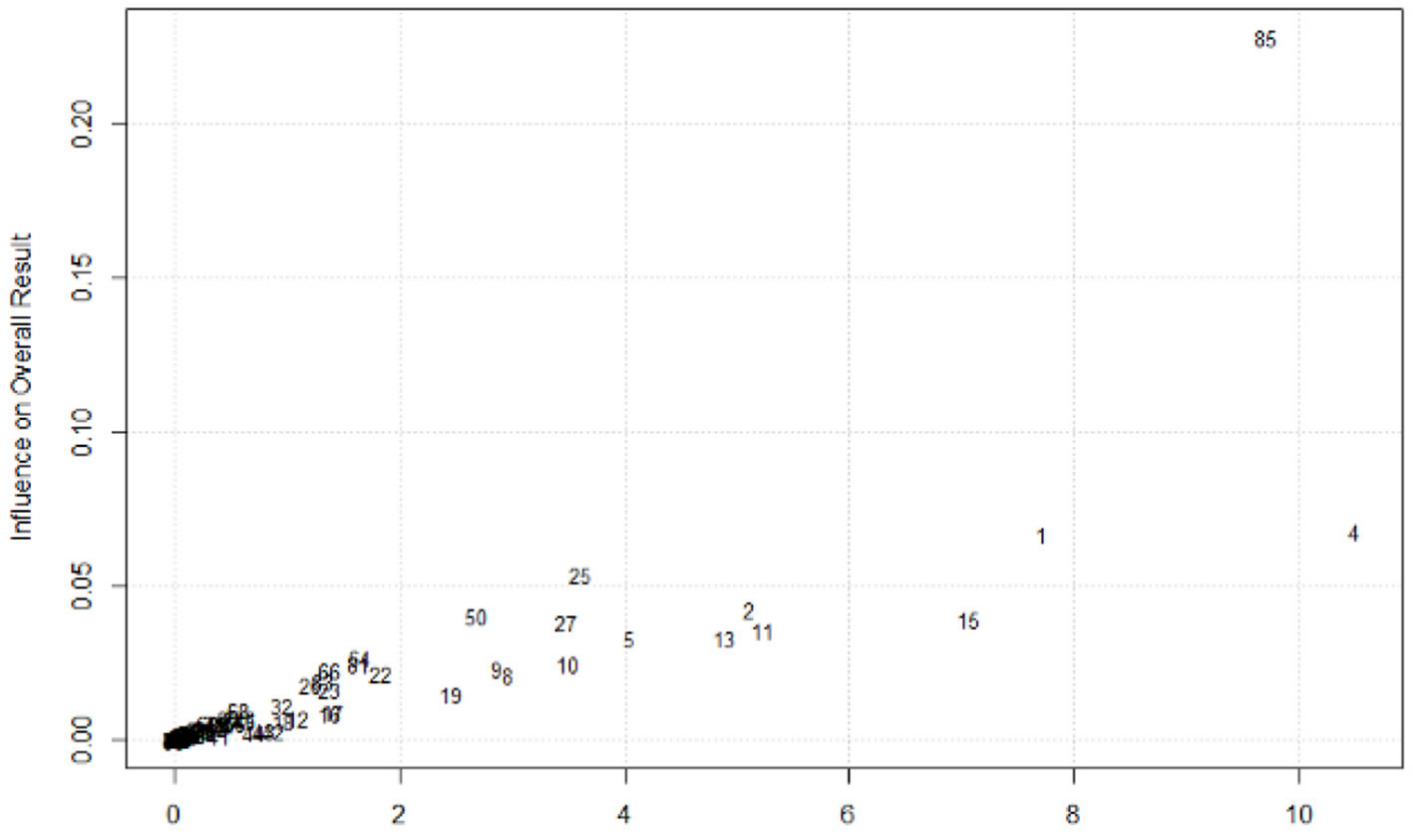
Baujat plot. Notes: each number represents one study included in this research.

As Figure 2 shows, the results of the study conducted by Wilksch et al. (2019) were found to be significantly different from those of the others. Thus, this study was removed from subsequent data analyses as an outliner.

### Overall Analysis

**Figure 3:**
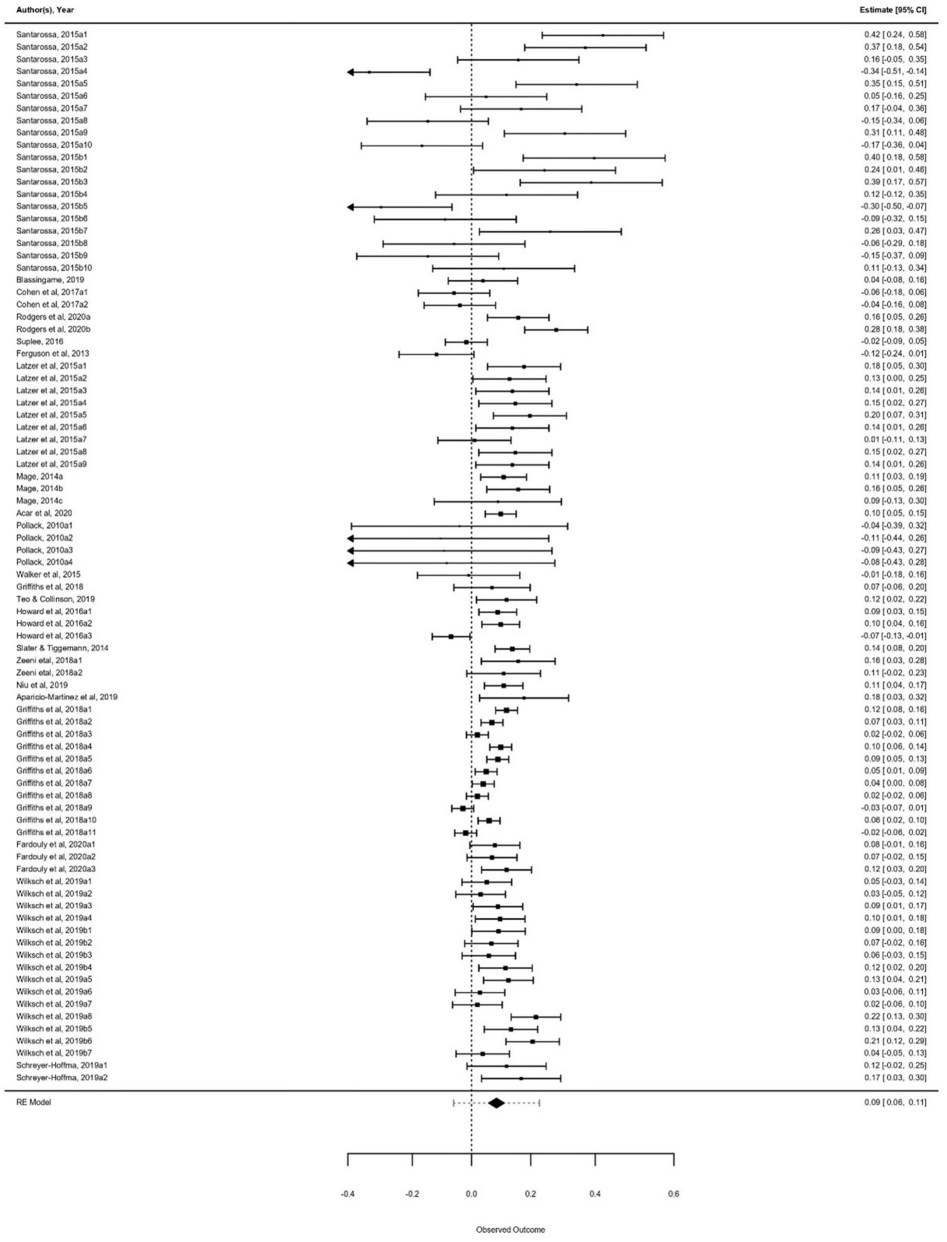
Forest plot for all samples. Notes: The horizontal lines show 95% confidence interval; the diamond represents the point estimate and confidence interval of the pooled effect size.

From the 22 studies that examined the correlation between SNS and disordered eating behaviors, 87 effect sizes were observed, ranging from −0.35 to 0.45. Significant heterogeneity existed among the effect sizes [*Q*_(df=85)_ = 304.27, *p* < 0.000] which suggested a need for further moderator analysis to explain heterogeneity. The Forest plot for all samples is presented in Figure 3.

### Moderator Analysis

Table 1 shows the moderator analysis results for the correlation between SNS and disordered eating behaviors. There were three statistically significant factors: BMI, with *F*_(1,34)_ = 6.080 (*p* = 0.019), Sample source, with *F*_(3,82)_ = 2.876 (*p* = 0.041) and Survey methods, with *F*_(1,84)_ = 5.253 (*p* = 0.024). Although two factors—Region factors, with *F*_(1,84)_ = 2.776 (*p* = 0.099) and Measure of eating, with *F*_(5,80)_ = 2.252 (*p* = 0.057)—were found to approach significance in the moderator analysis, these two factors' effects were >0.05, indicating that neither was significant. The rest of the moderators were found non-significant.

**Table 1:** Moderator analyses for studies reporting the correlation between SNS and disordered eating behaviors.

**Moderator variables**	**^#^Studies**	**^#^ES**	**β_0_ (95% CI)**	**ESr**	**β_1_ (95% CI)**	***F(df1,df2)***	**Level 2 variance**	**Level 3 variance**
Publication year	27	87	−11.707 (−32.051; 8.638)		0.006 (−0.004; 0.016)	1.329 (1, 84)	0.005^***^	0.001
Age	18	36	0.126 (−0.023; 0.276)		−0.003 (−0.011; 0.005)	0.523 (1, 34)	0.002^**^	0.002
BMI	13	37	0.746 (0.199; 1.292)^**^		−0.032(−0.058; −0.006)^*^	6.080 (1, 34)^*^	0.001	0.002
Percent of male	11	39	0.089 (0.057; 0.121)		−0.007 (−0.058;0.045)	0.065 (1, 84)	0.005^***^	0.001
Percent of college	14	37	0.107 (0.071; 0.144)		0.001 (−0.000; 0.003)	2.194 (1, 32)	0.007^***^	0.000
Percentage of white	14	23	0.049 (−0.049; 0.147)		0.008(−0.136; 0.152)	0.014 (1, 23)	0.002^**^	0.003
Publication type						0.113(1, 84)	0.005^***^	0.001
Journal	21	59	0.089 (0.057; 0.121)	0.088				
Thesis	6	28	−0.012 (−0.085; 0.060)	−0.0120	−0.101			
Region						2.776 (1, 84)^∧^	0.005^***^	0.000
Western	22	73	0.077 (0.050; 0.104)^***^	0.0768^***^				
Eastern	5	14	0.052 (−0.010; 0.114)	0.0520	−0.025			
Survey methods						5.253 (1, 84)^*^	0.004^***^	0.000
Paper–and-pencil	16	43	0.114 (0.081; 0.147)^***^	0.114^***^				
Online	11	44	−0.055 (−0.102; −0.007)^*^	−0.055^*^	−0.169			
Sample source						2.876 (3, 82)^*^	0.005^***^	0.000
University sample	12	35	0.089 (0.049; 0.129)^***^	0.089^***^				
Children and adolescent sample	7	29	0.035 (−0.023; 0.092)	0.035	−0.054			
Clinical sample	1	4	−0.171 (−0.379; 0.038)	−0.169	−0.26			
Other sample	7	19	−0.039 (−0.099; 0.021)	−0.039	−0.128			
SNS use measure						0.105 (3, 82)	0.005^***^	0.001
Duration	13	51	0.084 (0.040; 0.127)	0.084				
Frequency	10	32	0.010 (−0.052; 072)	0.010	0.074			
Intensity	2	2	−0.014 (−0.171; 0.143)	−0.014	−0.098			
Mixed	2	2	−0.024 (−0.170; 0.122)	−0.024	0.108			
SNS type							0.005	0.002
Image-based	10	34	0.070 (0.027; 0.112)^*^	0.070				
Non image–based	13	31	−0.234 (−0.334; −0.134)^***^	−0.223	−0.304			
General	14	22	0.044(−0.021.108)	0.044	−0.026			
Type of disordered eating						0.712(4, 82)	0.006^***^	0.000
Combined disordered eating behavior	22	54	0.077 (0.047; 0.106)^***^	0.077				
Binge eating	4	18	0.028 (−0.033; 0.090)	0.028	−0.049			
Driving for thinness	3	5	0.005 (−0.086; 0.097)	0.005	−0.072			
Bulimia	2	4	0.025 (−0.080; 0.131)	0.025	−0.052			
Dietary restraint	5	6	0.069 (−0.025; 0.164)	0.069	−0.008			
Measure of eating						2.252(5, 80)^∧^	0.005^***^	0.000
EAT-26	13	25	0.109 (0.068; 0.150)^***^	0.109				
Project Eat III–Eating behavior questions	2	10	−0.005 (−0.099; 0.089)	−0.005	−0.114			
Eating disorder inventory	4	11	−0.018 (−0.091; 0.055)	−0.018	−0.127			
Dutch eating behavior questionnaire	3	3	0.073 (−0.033; 0.180)	0.073	−0.036			
The EDE-Q	9	28	−0.058 (−0.109; −0.006)^*^	−0.058	−0.167			
Others	3	10	0.007 (−0.064; 0.078)	0.007	−0.102			

# *Studies, Number of studies; #ES, Number of effect sizes; CI, Confidence interval; Level 2 variance, Variance in effect sizes within studies; Level 3 variance, Variance in effect sizes between studies*.

∧ *p < 0.1*.

* *p < 0.05*.

** *p < 0.01*.

*** *p < 0.001*.

### Publication Bias

**Figure 4:**
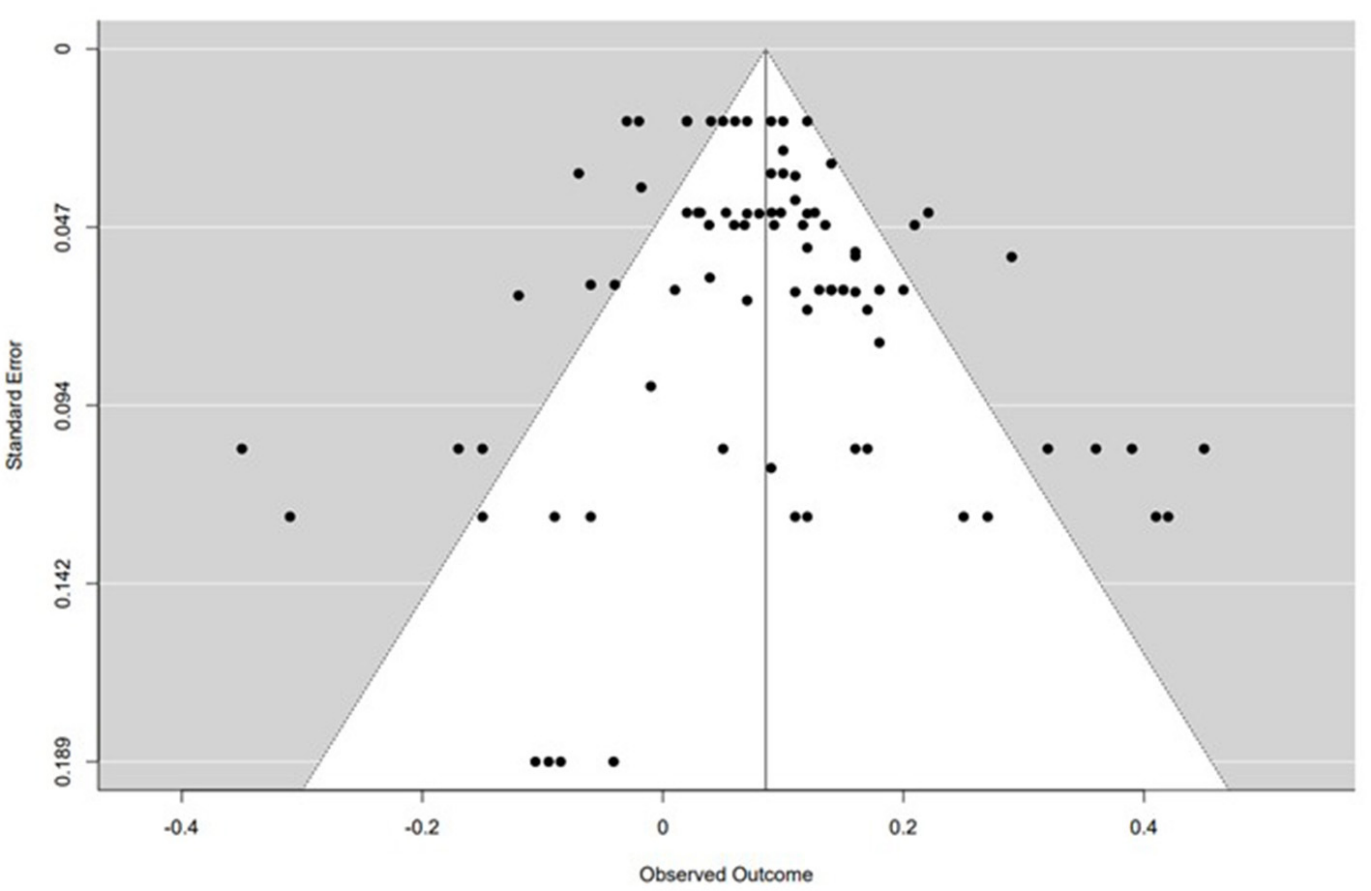
Funnel plot. Notes: Black circles are the studies included in the meta-analysis.

The Rank Correlation Test for Funnel Plot Asymmetry indicated no publication bias for the correlation between SNS and disordered eating behavior (Kendall's tau = 0.050, *p* = 0.530). The funnel plot is presented in Figure 4.

## Discussion

The current study was intended to expand upon previous work by adopting a three-level meta-analysis model to analyze the association between SNSs and disordered eating behaviors. Analysis in this study revealed a weak but significant positive correlation between the use of SNSs and disordered eating behaviors, in line with the results of several previous studies (Mabe et al., 2014; Latzer et al., 2015; Santarossa, 2015; Aparicio-Martinez et al., 2019; Niu et al., 2019; Teo and Collinson, 2019; Blassingame, 2020b; Rodgers et al., 2020). Considering that the focus of analysis in this study was the influence of frequency and duration of SNS usage on eating disorders, combined with the social-cultural model and self-objectification theory, the high frequency and long-term use of SNSs might indeed lead people to participate regularly in social comparisons (Ho et al., 2016). Excessive immersion in the appearance comparisons in the surrounding environment may make it more difficult for people to generate a positive body image, and may also lead people to suffer from stress relating to appearance and body shape, leading in turn to various mental illnesses, including disordered eating behaviors (Tylka and Sabik, 2010).

Moderation models help to understand if other variables explain the strength of relationship between two variables. This study therefore assessed several potential moderators, hoping to gain a clearer understanding of the inconsistency of findings in the literature as to whether and to what extent SNS usage is related to disordered eating behaviors. Moderator analyses showed that the sample source, survey method, and mean BMI of the sample were the significant moderators, which may explain the individual differences of the previous findings.

As for the sample source, university students were the main contributors to the discrepancies in disordered eating behaviors associated with SNS usage, compared with children, adolescents, clinical samples, and other samples. College students are the main contributors to studies investigating social media use (Zhang and Leung, 2015). For university students, social media are not only communication tools, but also form an important part of their daily routines (Madge et al., 2009). Regular use of SNSs may amplify the impact of SNSs on university students' lives, either positively or negatively (Gosling and Mason, 2015). At the same time, research has shown that some college students often use certain SNSs (such as Snapchat and Instagram) to make or receive appearance-related comparisons or comments (Verduyn et al., 2015). In other words, the purpose and of college students' use of social networks seems to vary the impact of the social networks has on their body image, which may further lead to further differences in their disordered eating habits. Therefore, when investigating the social media and disordered eating behaviors of college students, the interactions between SNSs usage and disordered eating behaviors are not in close accordance.

The survey methods are likely to also adjust the association between disordered eating behaviors and SNSs usages between different subgroups, because sometimes measuring the same variable through different methods (such as the pen-and-paper and online methods mentioned in this article) may yield different results (Moessner et al., 2015). Many tools for measuring eating disorder behaviors, including some commonly used tools, such as EAT by Garner and Garfinkel (1979), and BULIT-R by Thelen et al. (1991), have no dedicated online versions. When comparing the results of these questionnaire surveys in the laboratory with those on the Internet, some studies found that the two are highly similar, but some studies have obtained the opposite results (Joinson, 1999; Rammstedt et al., 2004). Therefore, although online surveys can collect data more conveniently and quickly from various channels if the survey tool used is not a dedicated online version, the results obtained may be different from the data collected face-to-face.

As far as BMI is concerned, the results indicate that an increase in the average BMI of the sample is usually accompanied by a decrease in the tendency for people to suffer from eating disorders associated with SNS use. BMI has been shown to have a significant impact on an individual's eating behaviors (Burnette et al., 2018). Unlike the positive relationship between BMI and disordered eating behaviors claimed by most research, this result indicates that a higher level of BMI may decrease the possibility of people having irregular eating behaviors after using of SNSs for a long time or in high intensity (Goldschmidt et al., 2008). Since there are no existing research results that allow us to explain this mechanism, we can only deduce that people with higher BMI may avoid SNS-related social behaviors, and that they may thus be exposed to fewer negative impacts from SNS that may lead to disordered eating behaviors. Since the figures of people with a lower BMI are more in line with the ideal body shape in most cultural environments, they might be more inclined to participate in the body comparisons on SNSs and further internalize the ideal slimness value, which may result in a negative view of their body and lead in turn to disordered eating behaviors and attitudes. Similarly, Yao et al. (2021) argue that people with a low BMI are more likely to demonstrate restrained eating behaviors because they lack confidence in their body. It is therefore reasonable to infer that people with a lower BMI could be more vulnerable to disordered eating behaviors when participating in activities related to SNSs.

In conclusion, the present meta-analysis has offered a quantitative synthesis of the current state of knowledge on the relationship between SNS usage and disordered eating behaviors. Based on a three-level meta-analytic model and moderator analysis, the research has demonstrated that SNS use is significantly linked to disordered eating behaviors and attitudes, which might be altered by the sample source, survey method, and mean BMI of the sample. According to sociocultural theory and self-objectification theory, individuals who use SNSs frequently and intensively seems to be more likely to internalize the ideal value of slimness of their social and cultural environment through information in SNSs, and to take part in social comparisons related to appearance. On the other hand, SNS usage might encourage individuals to connect their values with their body shapes. Both these inferences suggest that SNS usage is likely to lead to body dissatisfaction and may indeed play a causal role in the development of disordered eating behaviors.

## Limitations and Future Research Directions

Some limitations should be considered when interpreting the current research. First, language was set to English when filtering the research. In addition, some gray literature (such as ongoing research) would not appear in the general search process. This may have caused some literature to be lost from analysis of this study. Therefore, future meta-analyses should include richer studies to conduct a more comprehensive and thorough analysis of related issues. Second, in addition to the proposed moderator, there might be other factors that affect the consistency of the research results, such as sexual orientation and the ways of using SNSs (Ryding and Kuss, 2019; He et al., 2020). As there were few relevant studies, the influence of these factors on the relationship between SNS and eating disorders could not be analyzed, which awaits future research to fill these gaps. Third, because not every study contains the potential moderators that this meta-analysis set, this mean that the moderator analysis results are not applicable to all the studies in the relevant field. It therefore remains an important direction for future research to include more factors when identifying the situations and mechanisms concerning whether and how SNS usage is related to individuals' body dissatisfaction.

Future research needs to explore the other popular and current forms of SNSs (i.e., Twitter, Instagram, and Pinterest) owing to the rapid development of SNS platforms (Duggan et al., 2015). Moreover, specific ways (active/passive) of using SNSs should also be considered when conducting future research, to enhance understandings of the mechanisms and situations affecting whether and how SNS usage is associated with disordered eating.

## Conclusion and Implications

The current meta-analysis revealed a small, positive correlation between frequent and intensive use of SNSs and disordered eating behaviors. In addition, the BMI of the sample, the source of the sample, and the survey method (paper-and-pencil or online) were identified as the moderators that may explain the inconsistent findings between SNSs usage and disordered eating.

The findings from this meta-analysis have several clinical implications. First, this study found a positive correlation between the use of SNS and disordered eating behaviors. Clinicians may therefore consider evaluating the influence of SNS use on patients' irregular eating behaviors during the intervention process for disordered eating behaviors, and intervene with a view to controlling the length and frequency of SNS use.

The current meta-analysis also draws attention to the importance of the proper usage of SNSs in preventing disordered eating behaviors. According to the sociocultural theory and objectification theories which may explain the underlying principles of SNS usage and their positive associations with disordered eating behaviors, in addition to the need to control the frequency and duration of use of SNSs, the overall aesthetic orientation of these media and the comments made in them by the others are what affect the consequences of using SNSs. Frequent social comparisons on social media may aggravate the conflicts between the glamorous social images that people see displayed on their homepages and their perceptions of themselves. The findings of this meta-analysis may therefore have several media-related implications. Relevant institutions are advised to regularly organize some positive campaigns on SNSs to encourage people to pay attention to personal characteristics other than mere appearance. The promotion of the positive use of SNSs, which has been connected to fewer negative outcomes such as eating disorders and more positive outcomes such as the formation of social bonds, is also recommended (Verduyn et al., 2017). It is hoped that the current research can draw attention to the need to create a positive and healthy network environment for netizens in which SNSs facilitate their communication and help to establish their social relationships.

## Data Availability Statement

The raw data supporting the conclusions of this article will be made available by the authors, without undue reservation.

## Author Contributions

YW and JZ contributed to the research design. YW and QL collected relevant articles, completed the coding, and drafted the manuscript. YW analyzed the data. JZ and CW carefully revised the manuscript. All authors read and approved the final version of the manuscript.

## Conflict of Interest

The authors declare that the research was conducted in the absence of any commercial or financial relationships that could be construed as a potential conflict of interest. The Reviewer XX declared a shared affiliation with several of the authors, JZ, YW, and QL, to the handling editor at time of review.

## Publisher's Note

All claims expressed in this article are solely those of the authors and do not necessarily represent those of their affiliated organizations, or those of the publisher, the editors and the reviewers. Any product that may be evaluated in this article, or claim that may be made by its manufacturer, is not guaranteed or endorsed by the publisher.

## Appendix A

Quality assessment.

**Table:**

**References**	**Q1**	**Q2**	**Q3**	**Q4**	**Q5**	**Q6**	**Total**	**Quality**
Santarossa (2015)	1	1	1	1	1	1	6	100%
Blassingame (2020a)	1	0	1	1	1	1	5	83.3%
Cohen et al. (2017)	0	0	1	1	0	1	4	66.7%
Rodgers et al. (2020)	1	0	1	1	1	1	5	83.3%
Suplee (2018)	1	0	1	1	1	1	5	83.3%
Ferguson et al. (2013)	0	0	1	1	1	1	4	66.7%
Latzer et al. (2015)	0	1	1	1	1	1	5	83.3%
Mabe et al. (2014)	0	0	1	1	1	1	4	66.7%
Acar et al. (2020)	0	0	1	1	1	1	4	66.7%
Pollack (2017)	1	0	1	1	1	1	4	66.7%
Walker et al. (2015)	1	1	1	1	1	1	6	100.0%
Griffiths et al. (2018a)	1	1	1	1	0	1	5	83.3%
Teo and Collinson (2019)	1	1	1	1	1	1	6	100.0%
Howard et al. (2017)	1	1	1	1	1	1	6	100.0%
Slater and Tiggemann (2014)	1	0	1	1	1	1	5	83.3%
Zeeni et al. (2018)	0	0	1	1	1	1	4	66.7%
Niu et al. (2019)	1	0	1	1	1	1	4	83.3%
Aparicio-Martinez et al. (2019)	0	1	1	1	1	1	5	83.3%
Griffiths et al. (2018b)	1	0	1	1	0	1	4	66.7%
Fardouly et al. (2020)	1	1	1	1	1	1	6	100.0%
Wilksch et al. (2019)	0	0	1	1	1	1	4	66.7%
Schreyer-Hoffman (2020)	1	0	1	1	1	1	4	83.3%

*Q1 Sampling method: Was it representative of the population intended in the study?*.

*Q2 Was a response rate mentioned within the study? (Respond no if response rate was below 60%)*.

*Q3 Was the measurement tool of disordered eating behaviors valid and reliable?*

*Q4 Was the data source primary or secondary?*.

*Q5 Was SNSs usage examined in the study in a valid and reliable way?*

*Q6 Was the relationship or association between disordered eating behaviors and SNSs explored?*
